# Quantitative trait loci at the 11q23.3 chromosomal region related to dyslipidemia in the population of Andhra Pradesh, India

**DOI:** 10.1186/s12944-017-0507-5

**Published:** 2017-06-13

**Authors:** Rayabarapu Pranavchand, Battini Mohan Reddy

**Affiliations:** Molecular Anthropology Group, Biological Anthropology Unit, Indian Statistical Institute, Hyderabad, Telangana, India

**Keywords:** Quantitative lipid traits, Lipoprotein metabolism, Dyslipidemia, Genetic association, Haplotype

## Abstract

**Background:**

Given the characteristic atherogenic dyslipidemia of south Indian population and crucial role of *APOA1, APOC3, APOA4* and *APOA5* genes clustered in 11q23.3 chromosomal region in regulating lipoprotein metabolism and cholesterol homeostasis, a large number of recently identified variants are to be explored for their role in regulating the serum lipid parameters among south Indians.

**Methods:**

Using fluidigm SNP genotyping platform, a prioritized set of 96 SNPs of the 11q23.3 chromosomal region were genotyped on 516 individuals from Hyderabad, India, and its vicinity and aged >45 years.

**Results:**

The linear regression analysis of the individual lipid traits viz., TC, LDLC, HDLC, VLDL and TG with each of the 78 SNPs that confirm to HWE and with minor allele frequency > 1%, suggests 23 of those to be significantly associated (*p* ≤ 0.05) with at least one of these quantitative traits. Most importantly, the variant rs632153 is involved in elevating TC, LDLC, TG and VLDLs and probably playing a crucial role in the manifestation of dyslipidemia. Additionally, another three SNPs rs633389, rs2187126 and rs1263163 are found risk conferring to dyslipidemia by elevating LDLC and TC levels in the present population. Further, the ROC (receiver operating curve) analysis for the risk scores and dyslipidemia status yielded a significant area under curve (AUC) = 0.675, suggesting high discriminative power of the risk variants towards the condition. The interaction analysis suggests rs10488699-rs2187126 pair of the *BUD13* gene to confer significant risk (Interaction odds ratio = 14.38, *P* = 7.17 × 10^5^) towards dyslipidemia by elevating the TC levels (β = 37.13, *p* = 6.614 × 10^5^). On the other hand, the interaction between variants of *APOA1* gene and *BUD13* and/or *ZPR1* regulatory genes at this region are associated with elevated TG and VLDL.

**Conclusion:**

The variants at 11q23.3 chromosomal region seem to determine the quantitative lipid traits and in turn dyslipidemia in the population of Hyderabad. Particularly, the variants rs632153, rs633389, rs2187126 and rs1263163 might be risk conferring to dyslipidemia by elevating LDLC and TC levels, while the variants of *APOC3* and *APOA1* genes might be the genetic determinants of elevated triglycerides in the present population.

**Electronic supplementary material:**

The online version of this article (doi:10.1186/s12944-017-0507-5) contains supplementary material, which is available to authorized users.

## Background

Genetic etiology of coronary heart disease (CHD) suggests that the traditional risk factors such as dyslipidemia, diabetes and hypertension have their own independent genetic architecture. With its direct role in the process of development of atherosclerosis, dyslipidemia is the primary cause for atherosclerotic cardiovascular deaths, ranked as number one cause of deaths in India. The underlying pathophysiology includes disrupted cholesterol transport system and lipoprotein metabolism. The *APOA1, APOC3, APOA4* and *APOA5* genes clustered in 11q23.3 human chromosomal region are predominantly expressed in the liver and intestine and are crucial in regulating lipoprotein metabolism and cholesterol homeostasis [1]. About 180 polymorphisms have been identified in this gene cluster region and a couple of those were found to be associated with elevated plasma triglycerides among the Caucasians [2]. A few of the conventional polymorphisms that belong to *APOA1* and *APOA5* have also been identified as associated with elevated triglycerides and low density lipoprotein (LDL) particle size within the families of familial combined hyperlipidemia [3, 4]. The candidate gene and genome wide association studies (GWAS) have identified several of these polymorphisms to be associated with serum lipid parameters across the ethnic groups [5, 6]. Particularly, rs964184 that is found associated with multiple blood metabolite traits is mostly replicated for its association with triglycerides and decreased HDL. Given the characteristic atherogenic dyslipidemia of Indians [7, 8] and the putative role of apolipoprotein genes in regulating the serum lipid levels, a few attempts were made to understand the patterns of association of single nucleotide polymorphisms (SNP) at this region with lipid traits [9–11]. While, two of these studies validated the association of GWAS identified variants with elevated triglycerides among the north Indians [10, 11], Shanker et al. [9] observed the association of conventional polymorphisms with elevated triglycerides, LDL cholesterol and APOA1 protein among the south Indians. However, no comprehensive attempts have been made in order to understand the role of variants at 11q23.3 region in regulating the serum lipid parameters among south Indians. The present study is an attempt to analyze the pattern of association of a prioritized set of 96 SNPs, representing *APOAI-CIII-AIV-AV* gene cluster region, with serum lipid traits in the population of Hyderabad, India.

## Methods

### The study design and population

In the present study, 516 individuals aged ≥45 years were recruited by conducting health camps in and around Hyderabad that represents a conglomeration of people from different parts of undivided state of Andhra Pradesh. The populations of Andhra Pradesh were observed to be genetically homogenous [12] and the subjects of present study are the native Telugu speakers. Data pertaining to age, sex and history of diabetes, hypertension and dyslipidemia were obtained through a detailed questionnaire. Blood pressure and anthropometric measurements were also recorded for all the participants at the time of recruitment. About 5-6 ml of fasting blood sample of each subject was collected peripherally by certified medical lab technicians. Clinical investigations were done for lipid profile and blood sugar at Tapadia diagnostic centre, Hyderabad, using Auto Analyzer.

### DNA isolation and SNP genotyping

DNAs were isolated from all the samples using phenol chloroform method [13] and quantified with the help of Thermo Scientific Varioskan™ Flash Multimode Reader using Quant-iT™ PicoGreen® dsDNA Assay Kit. In order to comprehensively genotype the variants at 11q23.3 chromosomal region, we gathered information on SNPs pertaining to this region from earlier candidate gene and sequencing studies and from databases particularly EBI-NHGRI GWAS database, HAPMAP and dbSNP. Given the key role of *BUD13* in splicing mechanism and *ZPR1* as essential protein for normal cell proliferation and signal transduction, we also included SNPs related to these regulatory protein coding genes, in addition to the SNPs at *APOAI-CIII-AIV-AV* genes clustered at 11q23.3 chromosomal region. A total of 130 SNPs, mostly studied through candidate gene and GWAS approaches, were subjected to Fluidigm D3 Assay design software [14] and a panel of 96 SNPs with high efficiency for genotyping was chosen. Genotyping was performed using fluidigm nanofluidic SNP genotyping system. Six 96.96 IFC chips were utilized for genotyping wherein the selected 96 SNPs were analyzed against 96 samples in each chip. These chips were thermal cycled and the endpoint fluorescent values were measured on Biomark™ system. Final sample wise genotype calls were obtained using Fluidigm SNP Genotyping Analysis software. However, of the 96 SNPs analyzed, only 78 SNPs were qualified for final analysis, after excluding SNPs with minor allele frequency < 1% and/or deviated from Hardy-Weinberg equilibrium (*P* < 0.001).

### Statistical methods

The descriptive statistical analysis of the background data on quantitative variables was done using MINITAB (version 17). Genotyping quality check and association analysis of alleles as well as haplotypes were done using PLINK [15]. Genotype-phenotype association analysis assuming different genetic models- dominant, co-dominant, recessive, over dominant and log-additive- and logistic regression analysis with covariates were performed using ‘SNPassoc’ package of R PROGRAM [16]. Cumulative genetic risk scores for each individual were obtained using Microsoft Excel. The predictive power (Reciever Operating Characteristic (ROC) curve) of these genetic risk scores in discriminating dyslipidemia is estimated using IBM SPSS (version 21).

## Results

### Clinical characteristics of the control cohort

The percentage of diabetes (defined as per American Diabetic Association criteria), dyslipidemia (defined as per NCEP ATP III Criteria) and hypertension (defined as per Association of Physicians of India (API)) are 35.5, 41.6 and 40.2, respectively, in the present cohort. A comparison in the mean levels of quantitative variables between the diabetic versus non diabetic, hypertensive versus non hypertensive and dyslipidemic versus nondyslipidemic groups is made and the results are provided in Additional file 1. It is observed that the mean BMI, FBS, SBP, TG and VLDL levels are significantly elevated in the diabetic group as compared to non-diabetic group. Similarly, mean levels of these traits along with DBP are significantly elevated in hypertensive individuals as compared to non hypertensive group. However, the mean BMI levels are not significantly different between dyslipidemic and non dyslipidemic individuals. On the other hand, except for HDLC, significantly elevated mean values of lipid traits are observed among the dyslipidemic individuals as compared to non dyslipidemic individual.

### Association of variants at 11q23.3 chromosomal region with total cholesterol and low density lipoprotein cholesterol

The linear regression analysis of lipid traits viz., TC, LDLC, HDLC, VLDL and TG with each of the 78 SNPs from the 11q23.3 chromosomal region suggests 23 of them to be significantly associated (*p* ≤ 0.05) with at least one of these quantitative traits (Table 1). Further, we compared the genotype wise mean levels of all the lipid traits assuming various genetic models and the best operating genetic model for each of these variants is provided in Additional file 2. Of the above 23 SNPs, 11 are associated with TC and ten with LDLC. Six SNPs viz., rs2187126, rs633389, rs1263163, rs5132, rs5081 and rs632153 are found to be commonly associated with increasing TC and LDLC levels in contrast to rs6589566, which is associated with decreasing TC and LDLC. Of these, rs2187126, rs633389 and rs1263163 are significant even after Benjamin Hochberg correction for multiple testing. Further, while rs17440396 is associated with increased TC, rs672143 and rs11216153 are associated with decreased TC. On the other hand, rs10488699, rs633867 and rs2849165 are specific to and associated with increasing LDLC. The SNP rs2854116 that is associated with decreased TC is also found associated with decreased TG and VLDL.

**Table 1:** Significant allelic associations of SNPs at 11q23.3 chromosomal region with the quantitative lipid traits obtained through linear regression analysis^a^

SNP	Total cholesterol		LDL cholesterol		Triglycerides		VLDL	
	β (95% CI)	*p* value	β (95% CI)	*p* value	β (95% CI)	*p* value	β (95% CI)	*p* value
rs11216126					−0.08 (−0.18 - -0.002)	0.056*		
rs11216129					−0.08 (−0.18 - -0.002)	0.055*		
rs17440396	0.10 (0.01–0.19)	0.022						
rs10488699			0.09 (0.002–0.18)	0.044				
rs17119975					−0.09 (−0.18 - -0.003)	0.043	−0.09 (−0.18 - -0.003)	0.043
**rs2187126**	**0.18 (0.09–0.27)**	**6.29 × 10** ^**−5**^	**0.18 (0.09–0.27)**	**5.55 × 10** ^**−5**^				
rs1942478					−0.11 (−0.20 - -0.02)	0.017	−0.11 (−0.20 - -0.19)	0.017
rs4417316					−0.10 (−0.19 - -0.01)	0.025	−0.10 (−0.19 - -0.01)	0.029
rs6589566	−0.10 (−0.23 - -0.05)	0.0018	−0.15 (−0.25 - -0.06)	0.0007				
**rs633389**	**0.18 (0.08–0.27)**	**0.0001**	**0.21 (0.12–0.30)**	**4.75 × 10** ^**−6**^				
rs633867			0.09 (0.002–0.18)	0.044				
rs672143	−0.01 (−0.18 - -0.008)	0.033			−0.09 (−0.18 - -0.003)	0.042		
rs6589567					0.08 (−0.005–0.17)	0.067*	0.08 (−0.005–0.17)	0.067*
**rs1263163**	**0.30 (0.21–0.38)**	**6.24 × 10** ^**−11**^	**0.28 (0.20–0.37)**	**2.73 × 10** ^**−10**^				
rs2849165			0.09 (0.002–0.1 8)	0.045				
rs2854117					0.10 (0.01–0.19)	0.024	0.10 (0.01–0.19)	0.026
rs2854116	−0.10 (−0.20–0.003)	0.044			−0.11 (−0.21 - -0.01)	0.021	−0.11 (−0.21 - -0.01)	0.024
rs5132	0.09 (−0.0001–0.018)	0.051*	0.10 (0.01–0.19)	0.027				
rs11216153	−0.009 (−0.18 - -0.005)	0.038						
rs5081	0.13 (0.04–0.22)	0.003	0.13 (0.04–0.22)	0.0034	0.09 (0.003–0.18)	0.042	0.09 (0.002–0.18)	0.043
rs5072					0.09 (0.0002–0.18)	0.049		
rs632153	0.13 (0.03–0.22)	0.006	0.11 (0.02–0.20)	0.0139	0.11 (0.02–0.20)	0.014	0.11 (0.02–0.20)	0.015

β-Standardized linear regression coefficient, Blank cell – Non significant, bold font indicates significant after Benjamin Hochberg correction

*Significant after adjusting for covariates

^a^Results for lone SNP (rs918144(C) that belong to *BUD13* gene) associated with HDLC are not given in the table but described with text

Except for rs2849164, the allelic association patterns of SNPs with TC and LDLC are similar after adjusting for covariates - age, sex and BMI (Additional file 3). With exception to rs5132 and rs11216153, the allelic associations of SNPs with TC showed similar effects in their genotypic mean values before and after adjusting for covariates. However, the variant rs672143 did not show any significant genotypic association after adjusting for the covariates. With respect to LDLC, the SNPs significant in the allelic association analysis exhibited similar effect in the genotypic association analysis. The rs633867 and rs5132 which showed significant genotype wise mean difference under over-dominant model are not significant after adjusting for covariates.

### Association of variants at 11q23.3 chromosomal region with triglycerides and very low density lipoproteins

A total of eight variants that showed similar association with TG and VLDL, rs17119975, rs1942478, rs4417316, and rs2854116 are associated with decreased levels and rs6589567, rs2854117, rs5081 and rs632153 are associated with increased levels of these lipid traits. Additionally, rs672143 is associated with decreased TG albeit not significant after adjusting for covariates. Further, rs5072 is associated only with increased TG. The association pattern remained same for the rest of SNPs after adjusting for covariates age, sex and BMI. Although two more SNPs, rs11216126 and rs11216129, turned out to be significantly associated with decreased TG after adjusting for the covariates, we did not observe significant association of the genotypes of these two variants with mean TG levels. In contrast, rs6589567 that turned out to be significantly associated with TG and VLDL after adjusting for the covariates showed significant genotypic mean differences under over dominant model. Overall, the genotype wise mean values showed significant heterogeneity for the nine TG associated SNPs and eight VLDL associated SNPs, and this pattern persists even after adjusting for covariates.

### The lone SNP associated with high density lipoprotein cholesterol

Only a single intronic variant rs918144(C) that belong to *BUD13* gene is found to be associated with elevated HDLC levels (β = 0.09(0.005–0.18); *p* = 0.038) and remained significant (β = 0.103 (0.01–0.19); *p* = 0.028) in covariate adjusted analysis. The genotype wise mean HDLC levels significantly varied among the genotypes/individuals under over dominant model (CT vs TT or CC; *p* = 0.035), even after adjusting for covariates (*p* = 0.039).

### Association of haplotypes at 11q23.3 chromosomal region with quantitative lipid traits

Using the Gabriel et al. [17] haplotype block definition criteria we identified 15 haplotype blocks in this chromosomal region labeled from H1 to H15 (Additional file 4). The test for their association with quantitative lipid traits using linear regression identified eight haplotypes belonging to four blocks i.e., H4, H6, H8, H14 to be significantly associated (*p* < 0.05) with LDL cholesterol while five of those were also associated with total cholesterol (Table 2). Of these, haplotype AT that belongs to H6, TT and TC that belongs to H8, and TGG that belongs to H14 haplotype blocks were found to be associated with elevated lipid traits. Except for GC haplotype that belong to H6, the pattern of association of other haplotypes remained significant even after adjusting for covariates age, sex and BMI.

**Table 2:** Significant haplotypes at 11q23.3 chromosomal region associated with LDL Cholesterol and Total cholesterol obtained through linear regression analysis

SNPs in Haplotype block	Haplotype	Frequency	LDL Cholesterol		Total cholesterol	
			β (95% CI)	*p* value	β (95% CI)	*p* value
rs623908, rs664059	AC	0.21	−6.06 (−11.1, −1.0)	0.018		
rs6589566, rs2075290	GC	0.20	−5.22 (−10.4, −0.1)	0.047*		
	GT	0.05	−18.4 (−28.2, −8.6)	0.0002	−28.3 (−39.3, −17.3)	7.39 × 10^−07^
	AT	0.74	7.97 (3.5, 12.4)	0.0005	8.44 (3.3, 13.5)	0.0012
rs633389, rs633867	TT	0.05	9.68 (0.5, 18.8)	0.038		
	TC	0.10	16.5 (9.1, 23.9)	1.47 × 10^−05^	16.4 (7.9, 24.9)	0.0001
	CC	0.84	−13.1 (−18.7, −7.5)	5.25 × 10^−06^	−12.6 (−19.0, −6.2)	0.0001
rs5132, rs5128, rs11216153	TGG	0.039	17.7 (6.2, 29.2)	0.0027	19.1 (5.9, 32.3)	0.0047

* Not significant after adjusting for covariates age, sex and BMI

A common pattern of association with triglycerides and VLDL is observed for five haplotypes that belong to H5, H6, H9 and H14 blocks (Table 3). Among these, TC haplotype belong to H5 and CCG haplotype that belong to H14 were found to be associated with elevated levels of these traits. The association pattern remained same after adjusting for the covariates except that CC haplotype that belong to H9 turns out to be significantly associated with decreased VLDLs after adjusting for the covariates. Further, only the CC haplotype of H1 block is significantly associated with HDLC and remained same even adjusting for the above covariates.

**Table 3:** The haplotypes that are significantly associated with Triglycerides and VLDL

SNPs in Haplotype block	Haplotype	Frequency	Triglycerides		VLDL	
			β (95% CI)	*p* value	β (95% CI)	*p* value
rs1942478, rs4417316	GT	0.34	−14.9 (−29.0, −0.8)	0.039	−2.99 (−5.8, −0.2)	0.038
	TC	0.64	16.8 (2.9, 30.7)	0.018	3.31 (0.5, 6.1)	0.02
rs6589566, rs2075290	GT	0.05	−45.3 (−78.2, −12.4)	0.007	−9.2 (−15.8, −2.6)	0.006
rs1160038, rs6589567	CC	0.30	−14.5 (−28.9, −0.1)	0.048	−2.83 (−5.69, 0.03)	0.053^$^
rs5132, rs5128, rs11216153	CCG	0.36	14.6 (0.5, 28.7)	0.043	2.92 (0.1, 5.7)	0.043

^$^Significant only after adjusting for covariates age, sex and BMI

### Association of variants at 11q23.3 chromosomal region with dyslipidemia

The logistic regression analysis of variants at this chromosomal region against dyslipidemia observed 10 variants to be uniquely associated at *p* ≤ 0.05 (Table 4). Of these variants, rs17440396, rs10488699 and rs2187126 are associated with increased risk towards dyslipidemia and belong to *BUD13* regulatory gene, while rs6589566 is risk reducing and belongs to *ZPR1* regulatory gene. This pattern of association remained same in the logistic regression analysis with covariates. On the other hand, of the four SNPs that belong to *APOA5*-*APOA4* intergenic region and associated with dyslipidemia rs633389, rs1263163 and rs1263171 showed increased risk towards the disease and rs672143 decreased risk. Further, of the two upstream variants from *APOC3* (rs2854116) and *APOA1* (rs632153) that were associated with the disease, only *APOA1* is risk conferring towards dyslipidemia.

**Table 4:** Significant allelic association of SNPs at 11q23.3 chromosomal region with dyslipidemia: Odds ratios from logistic regression of dyslipidemia on variant alleles, before and after adjusting for age, sex and BMI

SNP ID	Gene/Functional relevance	Alleles Minor/Major	Minor Allele Frequency		Unadjusted		Adjusted for Age, Sex and BMI	
			Cases	Controls	OR	*p*-value	OR	*p*-value
rs17440396	*BUD13*/Intronic	**A**/G	0.24	0.18	1.4(1.04–1.98)	0.025	1.5(1.08–2.18)	0.015
rs10488699		**A**/G	0.23	0.17	1.4(1.03–1.98)	0.031	1.5(1.04–2.03)	0.029
rs2187126^a^		**G**/A	0.19	0.09	2.2(1.50–3.25)	4.07 × 10^−5^	2.3(1.52–3.41)	6.04 × 10^−5^
rs6589566	*ZPR1*/Intronic	G/A	0.20	0.29	0.6(0.46–0.86)	0.003	0.7(0.5–0.91)	0.009
rs633389^a^	*APOA5*-A4/Intergenic	**T**/C	0.22	0.12	2.1(1.47–3.02)	3.72 × 10^−5^	2.0(1.41–2.87)	0.0001
rs672143		G/A	0.00	0.03	0.1(0.01–0.63)	0.002	0.1(0.01–0.72)	0.022
rs1263163^a^		**A**/G	0.30	0.14	2.6(1.86–3.57)	7.16 × 10^−9^	3.1(2.15–4.52)	2.19 × 10^−9^
rs1263171		**A**/G	0.48	0.40	1.4(1.07–1.82)	0.013	1.3(1.03–1.72)	0.024
rs2854116	*APOC3*/upstream 2 kb	A/G	0.40	0.48	0.7(0.54–0.97)	0.032	0.7(0.56–0.98)	0.038
rs632153	*APOA1*/upstream 2 kb	**T**/G	0.04	0.02	2.2(1.04–4.84)	0.035	2.4(1.08–5.22)	0.030

Bold indicates risk increasing nature of the minor allele

^a^Significant after correction for multiple testing

The genotypic association analysis results are congruent with allelic association results. The best genetic mode of action of these associated variants is selected as per the akaike information criterion and these results are furnished in the Additional file 5. Among the seven dyslipidemic risk conferring variants, four (rs2187126, rs633389, rs1263163 and rs632153) are associated under dominant genotypic model, two (rs10488699 and rs1263171) under recessive genotypic model and only one variant rs17440396 is associated under over dominant model. Further, while a log additive mode of risk reducing association is observed with dyslipidemia for rs6589566 and rs672143, a recessive mode of similar effect is observed in case of rs2854116. These genotypic association patterns remained same after adjusting for age, sex and BMI as covariates.

**Fig. 1:**
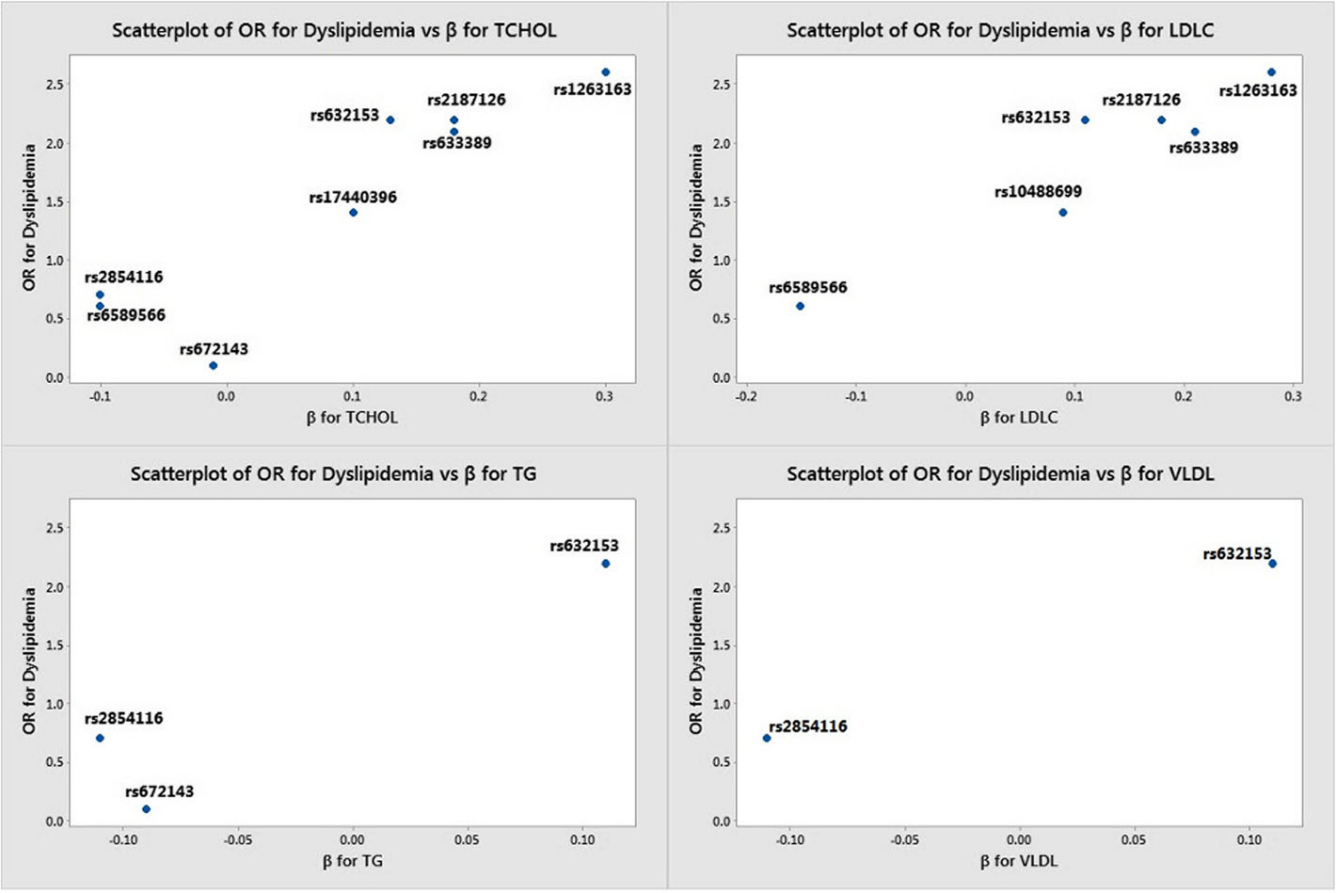
Effects of variants at 11q23.3 chromosomal region against dyslipidemia with relative to each of the quantitative lipid traits

To evaluate the relative effects of these genetic variants towards dyslipidemia and quantitative lipid traits, we plotted the effect estimates for SNPs that are commonly associated with dyslipidemia and each of the lipid traits. The scatter plots (Fig. 1) clearly suggest four SNPs namely rs632153, rs633389, rs2187126 and rs1263163 to be risk conferring to dyslipidemia by elevating LDLC and TC levels in the present population. The most important finding from the plot is the variant rs632153, which is involved in elevating TC, LDLC, TG and VLDLs, probably play a crucial role in the manifestation of dyslipidemia.

### Cumulative risk score analysis for the variants associated with dyslipidemia

**Fig. 2:**
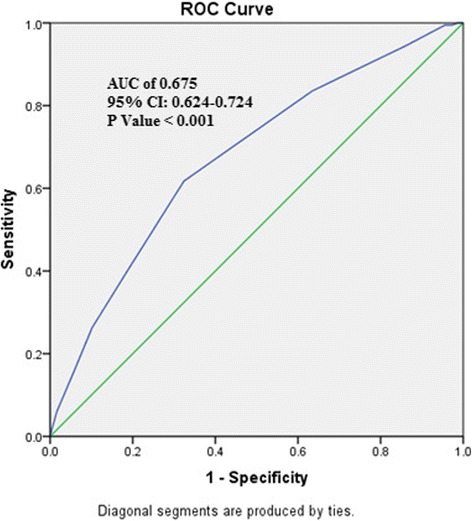
Receiver Operating Curve indicating the area under curve (AUC) and the discriminative power of risk scores towards dyslipidemia

In order to determine the combined risk effect of the associated genetic variants on dyslipidemia, we calculated the weighted mean proportion of the risk alleles for these SNPs by taking 2 for two risk alleles, 1 for one risk allele and 0 for no risk alleles with weights as relative log odds ratios of respective SNPs. Since the analysis requires 100% individual wise and SNP wise genotype data, we included only nine of the 10 SNPs on 430 individuals excluding rs2854116. The individual risk scores were multiplied with nine (number of SNPs used in the analysis) to obtain cumulative risk scores. The individuals with these cumulative risk scores ranging between 2.01 and 14.29 were then grouped into eight risk categories (Additional file 6). Given, the relatively low frequency of individuals with risk scores 2 to 5 and 11–14.29, these were merged into risk categories 1 and 8, respectively. With reference to risk category one, we computed odds ratios for the remaining seven risk categories. Although an increasing trend of odds ratio is observed with increasing risk score (Additional file 7), the OR values are significant (*p* < 0.05) only for risk categories 4 and above. In order to gauge the discriminative power of these variants for risk prediction towards dyslipidemia based on the risk scores, we constructed the ROC (receiver operating curve) plot (Fig. 2) for the risk scores and dyslipidemia status, which yielded area under curve (AUC) = 0.675, 95% CI 0.624–0.724 and *p* = 0.001, suggesting high discriminative power of the risk variants. Given these highly significant results, the observed AUC probably indicates that this study has substantial power to confer these genetic variants as predictors of risk for dyslipidemia.

### Pair wise SNP-SNP interaction

In order to understand the epistatic nature of these SNPs, we performed pair-wise interaction analyses for all the possible SNP pairs against dyslipidemia and quantitative lipid traits and found rs10488699-rs2187126 pair of the *BUD13* gene to confer significant risk (Interaction odds ratio = 14.38, *P* = 7.17 × 10^5^) towards dyslipidemia. This pair of SNPs is also found to significantly elevate TC levels (β = 37.13, *p* = 6.614 × 10^5^). However, we did not find any significant SNP-SNP interactions associated with LDLC and HDLC. Among the interactions associated with elevated TG and VLDL, we found rs5081 and rs632153 that belong to *APOA1* gene and commonly influenced by the BUD13 and ZPR1 regulatory genes at this region (Additional file 8).

## Discussion

Over three-fourth of general adult population in India is abnormal for at least one of the lipid traits, which are the important risk factors for CAD. Irrespective of the definition criteria used in the epidemiological literature, dyslipidemia is very high in India [18]. So far, the reported prevalence rates were based on populations studied from northern, central or extreme southern parts of India. The present study from Hyderabad with estimated incidence of 41.6% dyslipidemia broadly represents about 7.75 million people of south central India, as well as the characteristic atherogenic dyslipidemia of Asian Indians.

In the present study, we observed variants that belong to *BUD13*, *ZPR1* genes and *APOA5-APOA4* intergenic region as commonly associated with TC/LDLC and in turn with dyslipidemia. It implies that the risk towards dyslipidemia in the study population could be a consequential action of these variants which is implicated by the high discriminative power of the risk scores (illustrated by the AUC of 0.675 with 95% CI 0.624–0.724 and *p* value <0.001), suggesting that these genetic variants might be useful predictors of risk for dyslipidemia. On the other hand, seven variants across the 11q23.3 chromosomal region are observed to show common effects towards triglycerides and VLDLs. While significantly elevated levels of TG and VLDLs are regulated by the variants of *APOA1* and *APOC3* genes, the variants of regulatory genes and *APOA5-APOA4* intergenic region are associated with decreased levels of triglycerides. Therefore, it may be hypothesized that the variants of *APOA1* and *APOC3* might confer to hypertriglyceridemia, thereby leading to characteristic feature of atherogenic dyslipidemia among this south Indian population. However, a conventional polymorphism, rs5128 (SacI SNP) that belong to *APOA5* gene was found to be associated with elevated triglycerides in a south Indian population albeit not associated in our sample of Hyderabad [9]. Another study on western Indians found 3238C > G (rs5128) and -1131 T > C (rs662799) that belong to *APOC3* and *APOA5* genes, respectively, as associated with elevated levels of triglycerides and VLDLs [19]. Further, the most replicated GWAS variant rs964184 that wasidentified as associated with elevated triglycerides among Indians [10, 11] is also not evident in our study. The association of the variants of the regulatory genes with abnormal lipid traits is very much established among Caucasians [20] and has been replicated among other populations such as Chinese [21] and Japanese [22]. However, the variants of interest in the present study were not explored earlier among the Indians. Therefore, besides functional validation of these variants, they need to be replicated among other ethnic groups of India. Except for protective effects of rs918144 in elevating HDLC, we did not find any other variants associated with it, implying the negligible role of this chromosomal region in regulating HDLC levels. With its risk conferring nature towards dyslipidemia, rs632153 emerges as a prominent intronic variant of *APOA1* gene from our analysis. Conventional polymorphisms (−75G > A and +83 C > T) located in the regulatory regions of APOA1 gene were observed to be susceptible to CAD and elevated levels of TC, HDLC and TG [9, 23, 24]. Despite their prominent role in reverse cholesterol transport, these polymorphisms are not so far validated which needs to explored for the role in functional mechanism.

## Conclusion

In conclusion, the variants at 11q23.3 chromosomal region seem to determine the quantitative lipid traits and in turn dyslipidemia in the population of Hyderabad. Particularly, the variants rs632153, rs633389, rs2187126 and rs1263163 might be risk conferring to dyslipidemia by elevating LDLC and TC levels in the present population. These four SNPs exhibited a dominant mode of genotypic association with dyslipidemia, which implies that the *BUD13, ZPR1* and *APOA5-APOA4* intergenic regions might have a direct role in regulating these traits through their pleiotropic effects. Further, the variants of *APOC3* and *APOA1* genes might be the genetic determinants of elevated triglycerides in the present population. We suggest confirmation of the observed characteristics of 11q23.3 chromosomal region in multi ethnic studies in India that are base on much larger sample sizes well as through a more focused chromatin level studies with subsequent functional validation.

## Additional files

Additional file 1: Table S1. Means of quantitative clinical variables among the diabetic, hypertensive and dyslipidemic subjects as compared to the normal individuals and the p- values for the significance of mean differences as reflected by t-test. (DOCX 12 kb)

Additional file 2: Table S2. Genotypic associations of variants at 11q23.3 chromosomal region represented under best genetic mode of action along with genotype wise mean levels for TC, LDLC, TG and VDLC. (DOCX 21 kb)

Additional file 3: Table S3. Significant allelic associations of variants at 11q23.3 chromosomal region with quantitative lipid traits after adjusting for covariates. (DOCX 13 kb)

Additional file 4: Table S4. Details of Haplotype blocks. (DOCX 12 kb)

Additional file 5: Table S5. Significant genotypic association of variants at 11q23.3 chromosomal region with dyslipidemia under different genetic models: Odds ratios from logistic regression analyses before and after adjusting for age, sex and BMI. (DOCX 16 kb)

Additional file 6: Table S6. Percentage of Dyslipidemic and Non dyslipidemic individuals with cumulative risk scores for SNPs associated with dyslipidemia and results of logistic regression analysis of dyslipidemia on risk categories. (DOCX 11 kb)

Additional file 7: Plot of Odds ratios for cumulative risk score categories with reference to baseline category 1. (JPEG 28 kb)

Additional file 8: Table S7. Results of pair wise SNP-SNP interaction analysis; only significant pairs are represented in the table. (DOCX 11 kb)
