# Supplementary material for: Quantitative trait loci at the 11q23.3 chromosomal region related to dyslipidemia in the population of Andhra Pradesh, India
Source: Lipids Health Dis. 2017 Jun 13;16:116. doi: 10.1186/s12944-017-0507-5 (PMC5470178; doi:10.1186/s12944-017-0507-5)
Supplement: Supplementary file 1 — Means of quantitative clinical variables among the diabetic, hypertensive and dyslipidemic subjects as compared to the normal individuals and the p- values for the significance of mean differences as reflected by t-test. (DOCX 12 kb) [file 12944_2017_507_MOESM1_ESM.docx]

**Table S1 Means of quantitative clinical variables among the diabetic, hypertensive and dyslipidemic subjects as compared to the normal individuals and the p- values for the significance of mean differences as reflected by t-test.**

| **Quantitative variable** | **Non Diabetic**  **N = 298** | **Diabetic**  **N = 164** | **p- value** | **Non Dyslipidemic**  **N = 270** | **Dyslipidemic**  **N = 192** | **p- value** | **Non Hypertensive**  **N = 276** | **Hypertensive**  **N = 186** | **p- value** |
| --- | --- | --- | --- | --- | --- | --- | --- | --- | --- |
|  | **Mean (SD)** | |  | **Mean (SD)** | |  | **Mean (SD)** | |  |
| **FBS** | 78.0(9.2) | 132.9(54.3) | 0.0001* | 93.8(37.5) | 102.6(47.9) | 0.03* | 92.6(38.9) | 105.0(46.1) | 0.003* |
| **SBP** | 125.7(14.5) | 130.5(14.0) | 0.001* | 126.2(14.5) | 129.0(14.4) | 0.04* | 119.6(8.1) | 138.6(14.3) | 0.0001* |
| **DBP** | 83.0(9.4) | 84.4(8.8) | 0.129 | 83.0(9.5) | 84.2(8.9) | 0.19 | 79.8(6.0) | 88.8(10.4) | 0.0001* |
| **TCHOL** | 190.1(34.8) | 191.8(40.1) | 0.65 | 169.5(22.7) | 220.4(31.8) | 0.0001* | 188.3(33.8) | 194.2(40.6) | 0.103 |
| **TRGLY** | 146.1(71.1) | 189.3(147.4) | 0.001* | 134.6(60.6) | 199.0 (140.8) | 0.0001* | 151.6(85.2) | 176.1(131.4) | 0.02* |
| **HDLC** | 49.0(3.4) | 45.6(4.8) | 0.24 | 45.6(4.1) | 46.2(3.7) | 0.32 | 45.9(3.5) | 45.7(4.6) | 0.84 |
| **LDLC** | 114.9(31.1) | 111.0 (34.2) | 0.219 | 96.8(21.4) | 136.9(30.0) | 0.0001* | 112.9(29.6) | 114.4(35.9) | 0.65 |
| **VLDL** | 29.0(14.2) | 38.1(29.4) | 0.0001* | 27.0(12.2) | 39.5(28.2) | 0.0001* | 30.1(17.0) | 35.4(26.2) | 0.02* |
| **BMI** | 26.6(4.4) | 27.5(4.6) | 0.04* | 27.0(4.9) | 26.8(4.0) | 0.603 | 26.4(4.6) | 27.6(4.3) | 0.01* |

BMI – Body mass index, WC – Waist circumference, HC – Hip circumference, WHR – Waist to hip circumference ratio, FBS – Fasting blood sugar, SBP – Systolic blood pressure, DBP – Diastolic blood pressure, TC – Total cholesterol, TG – Triglycerides, HDLC – High density lipoprotein cholesterol, LDLC – Low Density lipoprotein cholesterol, VLDL – Very low density lipoproteins. *significant
