# Supplementary material for: Quantitative trait loci at the 11q23.3 chromosomal region related to dyslipidemia in the population of Andhra Pradesh, India
Source: Lipids Health Dis. 2017 Jun 13;16:116. doi: 10.1186/s12944-017-0507-5 (PMC5470178; doi:10.1186/s12944-017-0507-5)
Supplement: Supplementary file 2 — Genotypic associations of variants at 11q23.3 chromosomal region represented under best genetic mode of action along with genotype wise mean levels for TC, LDLC, TG and VDLC. (DOCX 21 kb) [file 12944_2017_507_MOESM2_ESM.docx]

**Table S2 Genotypic associations of variants at 11q23.3 chromosomal region represented under best genetic mode of action along with genotype wise mean levels for TC, LDLC, TG and VDLC**

| **SNP** | **Model** | **Genotype** | **No of Individuals** | **Unadjusted** | | | | **Adjusted for Age, Sex and BMI** | |
| --- | --- | --- | --- | --- | --- | --- | --- | --- | --- |
|  |  |  |  | **Mean(SE)** | | **p value** | | **Mean(SE)** | **p value** |
| **Total Cholesterol** | | | | | | | | | |
| rs17440396 | Over-Dominant | GG-AA | 283 | 186.7(2.1) | | 0.0032 | | 186.1(2.2) | 0.004 |
|  |  | AG | 169 | 197.0(2.8) | |  | | 197.2(2.8) |  |
| rs2187126 | Dominant | AA | 339 | 186.3(1.9) | | 7.03x10^-5^ | | 186.0(1.9) | 2.19x10^-5^ |
|  |  | AG-GG | 119 | 203.5(3.5) | |  | | 203.0(3.5) |  |
| rs6589566 | Recessive | AA-AG | 405 | 192.5(1.8) | | 0.0016 | | 192.2(1.8) | 0.0015 |
|  |  | AA | 41 | 173.7(4.9) | |  | | 173.7(4.8) |  |
| rs633389 | Over-Dominant | CC-TT | 340 | 186.1(1.8) | | 1.38x10^-6^ | | 185.6(1.8) | 3.8x10^‑6^ |
|  |  | CT | 111 | 205.3(4.0) | |  | | 205.3(4.0) |  |
| rs672143 | Dominant | AA | 426 | 191.9(4.9) | | 0.041 | |  | 0.055 |
|  |  | AG-GG | 16 | 172.0(4.9) | |  | |  |  |
| rs1263163 | Dominant | GG | 279 | 181.2(2.0) | | 1.4x10^-12^ | | 180.8(2.0) | 3.6x10^-12^ |
|  |  | AG-AA | 177 | 205.6(2.6) | |  | | 205.5(2.6) |  |
| rs2854116 | Recessive | GG-AG | 288 | 192.8(2.3) | | 0.023 | | 192.5(2.3) | 0.029 |
|  |  | AA | 86 | 182.2(3.2) | |  | | 181.2(3.3) |  |
| rs5081 | Log additive | AA | 415 | 189.6(1.7) | | 0.0025 | | 189.3(1.7) | 0.0028 |
|  |  | AT | 29 | 201.8(7.6) | |  | | 201.8(7.6) |  |
|  |  | TT | 1 | 340.0 | |  | | 340.0 |  |
| rs632153 | Co-Dominant | GG | 426 | 189.3(1.7) | | 0.004 | | 189.0(1.7) | 0.005 |
|  |  | GT | 29 | 209.4(8.7) | |  | | 209.4(8.7) |  |
| **Low Density Lipoprotein Cholesterol** | | | | | | | | | |
| rs1048669 | Log-additive | GG | 290 | 111.4(1.8) | 0.032 | | | 111.2(1.8) | 0.029 |
|  |  | GA | 150 | 115.8(2.8) |  | | | 115.8(2.8) |  |
|  |  | AA | 16 | 127.9(8.1) |  | | | 128.4(8.6) |  |
| rs2187126 | Dominant | AA | 339 | 109.7(1.6) | 8x10^-6^ | | | 109.4(1.6) | 1.07x10^-5^ |
|  |  | AG-GG | 115 | 125.1(3.2) |  | | | 125.1(3.2) |  |
| rs6589566 | Log-additive | AA | 261 | 117.2(2.0) | 0.0007 | | | 117.1(2.1) | 0.003 |
|  |  | AG | 144 | 110.8(2.5) |  | | | 110.6(2.3) |  |
|  |  | GG | 41 | 100.3(4.5) |  | | | 100.3(4.5) |  |
| rs633389 | Dominant | CC | 324 | 108.9(1.6) | 4.6x10^-7^ | | | 108.6(1.6) | 2.9x10^-6^ |
|  |  | CT-TT | 127 | 125.8(3.2) |  | | | 125.8(3.3) |  |
| rs633867 | Over-Dominant | CC-TT | 406 | 112.5(1.5) | 0.036 | | | 112.4(1.5) | 0.06 |
|  |  | CT | 48 | 122.8(5.4) |  | | | 122.7(5.5) |  |
| rs1263163 | Dominant | GG | 279 | 105.5(1.6) | 1.4x10^-11^ | | | 105.3(1.7) | 3.3x10^-11^ |
|  |  | GA-AA | 177 | 126.0(2.5) |  | | | 126.0(2.6) |  |
| rs2849165 | Over-Dominant | GG-AA | 246 | 107.2(1.8) | 4.9x10^-6^ | | | 107.0(1.8) | 6.9x10^-6^ |
|  |  | AG | 207 | 121.0(2.4) |  | | | 120.9(2.4) |  |
| rs5132 | Over-Dominant | CC-TT | 405 | 112.8(1.5) | 0.051 | | | 112.6(1.6) | 0.087 |
|  |  | CT | 40 | 123.3(6.0) |  | | | 123.2(6.0) |  |
| rs5081 | Co-dominant | AA | 415 | 112.6(1.5) | 1.3x10^-5^ | | | 112.4(1.5) | 7.9x10^-6^ |
|  |  | AT | 29 | 125.5(6.3) |  | | | 121.5(6.3) |  |
|  |  | TT | 1 | 258.0 |  | | | 258.0 |  |
| rs632153 | Co-dominant | GG | 426 | 112.5(1.5) | 0.014 | | | 112.3(1.5) | 0.021 |
|  |  | GT | 29 | 127.7(7.7) |  | | | 127.7(7.7) |  |
| **Triglycerides** | | | | | | | | | |
| rs17119975 | Dominant | TT | 198 | 173.6(9.8) | | | 0.036 | 173.1(9.9) | 0.031 |
|  |  | CT-CC | 256 | 152.4(4.6) | | |  | 151.4(4.6) |  |
| rs1942478 | Dominant | TT | 199 | 174.6(9.7) | | | 0.017 | 174.1(9.8) | 0.016 |
|  |  | GT-GG | 254 | 150.6(4.5) | | |  | 149.6(4.5) |  |
| rs4417316 | Log-additive | CC | 205 | 174.2(9.5) | | | 0.024 | 173.7(9.6) | 0.025 |
|  |  | CT | 177 | 155.1(5.5) | | |  | 153.9(5.5) |  |
|  |  | TT | 66 | 144.3(8.9) | | |  | 143.9(9.1) |  |
| rs672143 | Log-additive | AA | 426 | 164.4(5.2) | | | 0.044 | 163.7(5.3) | 0.048 |
|  |  | AG | 15 | 115.2(11.9) | | |  | 115.2(11.9) |  |
|  |  | GG | 1 | 53.0 | | |  | 53.0 |  |
| rs6589567 | Over dominant | CC-AA | 289 | 151.4(4.5) | | | 0.008 | 150.5(4.5) | 0.0069 |
|  |  | AC | 167 | 178.7(11.1) | | |  | 178.1(11.1) |  |
| rs2854117 | Dominant | GG | 133 | 141.8(5.5) | | | 0.01 | 139.9(5.4) | 0.005 |
|  |  | GA-AA | 310 | 170.5(6.8) | | |  | 170.1(6.9) |  |
| rs2854116 | Log-additive | GG | 122 | 172.2(10) | | | 0.023 | 171.9(10) | 0.015 |
|  |  | AG | 166 | 162.2(9.9) | | |  | 161.6(10) |  |
|  |  | AA | 86 | 136.2(5.6) | | |  | 133.3(5.2) |  |
| rs5081 | Dominant | AA | 415 | 158.8(4.3) | | | 0.029 | 158(4.4) | 0.020 |
|  |  | AT-TT | 30 | 203.1(44.9) | | |  | 203.2(44.9) |  |
| rs5072 | Log-additive | CC | 183 | 152.4(8.8) | | | 0.049 | 151.2(8.9) | 0.035 |
|  |  | CT | 190 | 162(6.6) | | |  | 161.5(6.7) |  |
|  |  | TT | 71 | 183.1(13.4) | | |  | 183.1(13.4) |  |
| rs632153 | Co-dominant | GG | 426 | 157.8(4.3) | | | 0.01 | 157.0(4.3) | 0.006 |
|  |  | GT | 29 | 210.2(46.3) | | |  | 210.2(46.3) |  |
| **Very Low Density Lipoprotein Cholesterol** | | | | | | | | | |
| rs17119975 | Dominant | TT | 198 | 34.5(1.9) | | | 0.039 | 34.5(1.9) | 0.037 |
|  |  | CT | 181 | 30.7(1.0) | | |  | 30.7(1.0) |  |
|  |  | CC | 78 | 29.2(1.7) | | |  | 29.2(1.7) |  |
| rs1942478 | Dominant | TT | 199 | 34.7(1.9) | | | 0.019 | 34.7(1.9) | 0.019 |
|  |  | TG | 189 | 30.6(1.0) | | |  | 30.4(1.0) |  |
|  |  | GG | 65 | 28.7(1.8) | | |  | 28.6(1.8) |  |
| rs4417316 | Log-additive | CC | 205 | 34.7(1.9) | | | 0.027 | 34.6(1.9) | 0.028 |
|  |  | CT | 177 | 30.7(1.1) | | |  | 30.5(1.1) |  |
|  |  | TT | 66 | 28.9(1.8) | | |  | 28.8(1.8) |  |
| rs6589567 | Over dominant | CC-AA | 289 | 30.2(0.9) | | | 0.007 | 30(0.9) | 0.0063 |
|  |  | AC | 167 | 35.7(2.2) | | |  | 35.6(2.2) |  |
| rs2854117 | Dominant | GG | 133 | 28.4(1.1) | | | 0.012 | 28(1.1) | 0.0069 |
|  |  | GA-AA | 310 | 34(1.3) | | |  | 33.9(1.3) |  |
| rs2854116 | Log-additive | GG | 122 | 34.4(1.9) | | | 0.026 | 34.3(2.0) | 0.018 |
|  |  | AG | 166 | 32.4(1.9) | | |  | 32.2(2.0) |  |
|  |  | AA | 86 | 27.3(1.1) | | |  | 26.7(1.0) |  |
| rs5081 | Dominant | AA | 415 | 31.6(0.8) | | | 0.03 | 31.5(0.8) | 0.02 |
|  |  | AT-TT | 30 | 40.4(8.9) | | |  | 40.4(8.9) |  |
| rs632153 | Co-dominant | GG | 420 | 31.5(0.8) | | | 0.01 | 31.3(0.8) | 0.007 |
|  |  | GT | 29 | 41.8(9.2) | | |  | 41.8(9.2) |  |
