# Supplementary material for: Quantitative trait loci at the 11q23.3 chromosomal region related to dyslipidemia in the population of Andhra Pradesh, India
Source: Lipids Health Dis. 2017 Jun 13;16:116. doi: 10.1186/s12944-017-0507-5 (PMC5470178; doi:10.1186/s12944-017-0507-5)
Supplement: Supplementary file 3 — Significant allelic associations of variants at 11q23.3 chromosomal region with quantitative lipid traits after adjusting for covariates. (DOCX 13 kb) [file 12944_2017_507_MOESM3_ESM.docx]

**Table S3 Significant allelic associations of variants at 11q23.3 chromosomal region with quantitative lipid traits after adjusting for covariates**

| **SNP** | **Total cholesterol** | | **LDL cholesterol** | | **Triglycerides** | | **VLDL** | |
| --- | --- | --- | --- | --- | --- | --- | --- | --- |
|  | **β (95% CI)** | **p value** | **β (95% CI)** | **p value** | **β (95% CI)** | **p value** | **β (95% CI)** | **p value** |
| rs11216126 |  |  |  |  | -0.09 (-0.18 - -0.001) | 0.048* |  |  |
| rs11216129 |  |  |  |  | -0.09 (-0.18 - -0.001) | 0.047* |  |  |
| rs17440396 | 0.10 (0.01 - 0.19) | 0.021 |  |  |  |  |  |  |
| rs10488699 |  |  | 0.09 (0.002 - 0.18) | 0.045 |  |  |  |  |
| rs17119975 |  |  |  |  | -0.09 (-0.18 - -0.006) | 0.037 | -0.09 (-0.18 - -0.005) | 0.038 |
| **rs2187126** | 0.18 (0.09 - 0.27) | 0.0001 | 0.18 (0.09 - 0.27) | 8.3 x10^-5^ |  |  |  |  |
| rs1942478 |  |  |  |  | -0.11 (-0.20 - -0.02) | 0.014 | -0.11 (-0.20 - -0.02) | 0.024 |
| rs4417316 |  |  |  |  | -0.10 (-0.20 - -0.01) | 0.021 | -0.10 (-0.19 - -0.01) | 0.029 |
| rs6589566 | -0.13 (-0.23 - -0.04) | 0.0034 | -0.15 (-0.24 - -0.06) | 0.0012 |  |  |  |  |
| **rs633389** | 0.17 (0.08 - 0.26) | 0.0002 | 0.20 (0.11 - 0.29) | 7.7 x10^-6^ |  |  |  |  |
| rs633867 |  |  | 0.09 (0.001 - 0.18) | 0.046 |  |  |  |  |
| rs672143 | -0.09 (-0.18 - -0.001) | 0.046 |  |  | -0.09 (-0.18 - 0.001) | 0.053 |  |  |
| rs6589567 |  |  |  |  | 0.09 (0.001 - 0.18) | 0.046 | 0.09 (0.0002 - 0.18) | 0.049 |
| **rs1263163** | 0.29 (0.20 - 0.38) | 1.7 x10^-10^ | 0.28 (0.19 - 0.37) | 6.4 x10^-10^ |  |  |  |  |
| rs2849165 |  |  | 0.08 (-0.002 - 0.18) | 0.058 |  |  |  |  |
| rs2854117 |  |  |  |  | 0.10 (0.01 - 0.19) | 0.023 | 0.10 (0.01 - 0.19) | 0.025 |
| rs2854116 | -0.10 (-0.20 - -0.006) | 0.037 |  |  | -0.12 (-0.22 - -0.02) | 0.019 | -0.11 (-0.21 - -0.01) | 0.022 |
| rs5132 | 0.09 (0.005 - 0.19) | 0.038* | 0.10 (0.01 - 0.20) | 0.020 |  |  |  |  |
| rs11216153 | -0.09 (-0.18 - -0.003) | 0.045 |  |  |  |  |  |  |
| rs5081 | 0.14 (0.05 - 0.23) | 0.0021 | 0.14 (0.05 - 0.23) | 0.0023 | 0.09 (0.006 - 0.19) | 0.036 | 0.09 (0.005 - 0.19) | 0.038 |
| rs5072 |  |  |  |  | 0.09 (0.002 - 0.18) | 0.044 |  |  |
| rs632153 | 0.13 (0.04 - 0.22) | 0.0037 | 0.12 (0.03 - 0.21) | 9.7 x10^-3^ | 0.11 (0.02 - 0.21) | 0.011 | 0.11 (0.02 - 0.20) | 0.012 |

β-Standardized linear regression coefficient, Blank cell – Non significant, * Significant only after adjusting for covariates, bold font indicates significant after Benjamin Hochberg correction
