# Supplementary material for: Quantitative trait loci at the 11q23.3 chromosomal region related to dyslipidemia in the population of Andhra Pradesh, India
Source: Lipids Health Dis. 2017 Jun 13;16:116. doi: 10.1186/s12944-017-0507-5 (PMC5470178; doi:10.1186/s12944-017-0507-5)
Supplement: Supplementary file 4 — Details of Haplotype blocks. (DOCX 12 kb) [file 12944_2017_507_MOESM4_ESM.docx]

**Table S4 Details of Haplotype blocks**

| **Haplotype Blocks** | **Nucleotide start position** | **Nucleotide end position** | **Region covered in BP** | **No of SNPs** | **SNPs** |
| --- | --- | --- | --- | --- | --- |
| H1 | 116746524 | 116749540 | 3.017 | 2 | rs11216126, rs11216129 |
| H2 | 116759884 | 116760974 | 1.091 | 3 | rs918143, rs3741301, rs3741300 |
| H3 | 116768388 | 116769225 | 0.838 | 2 | rs10790162, rs1263149 |
| H4 | 116769652 | 116771421 | 1.77 | 2 | rs623908, rs664059 |
| H5 | 116780747 | 116781585 | 0.839 | 2 | rs1942478, rs4417316 |
| H6 | 116781707 | 116782580 | 0.874 | 2 | rs6589566, rs2075290 |
| H7 | 116791863 | 116794060 | 2.198 | 3 | rs651821, rs662799, rs1787680 |
| H8 | 116796621 | 116796764 | 0.144 | 2 | rs633389, rs633867 |
| H9 | 116799466 | 116799960 | 0.495 | 2 | rs11600380, rs6589567 |
| H10 | 116804102 | 116807007 | 2.906 | 2 | rs1729408, rs1263167 |
| H11 | 116810292 | 116812658 | 2.367 | 2 | rs1263173, rs2727793 |
| H12 | 116813448 | 116814051 | 0.604 | 2 | rs7396851, rs2542063 |
| H13 | 116819862 | 116828729 | 8.868 | 11 | rs1268354, rs1263177, rs5095, rs2216311, rs2098453, rs2727789, rs2849176, rs2849174, rs2071523, rs2542051, rs595049 |
| H14 | 116832062 | 116834384 | 2.323 | 3 | rs5132, rs5128, rs11216153 |
| H15 | 116836867 | 116836968 | 0.102 | 2 | rs5072, rs2070665 |
