# Supplementary material for: Quantitative trait loci at the 11q23.3 chromosomal region related to dyslipidemia in the population of Andhra Pradesh, India
Source: Lipids Health Dis. 2017 Jun 13;16:116. doi: 10.1186/s12944-017-0507-5 (PMC5470178; doi:10.1186/s12944-017-0507-5)
Supplement: Supplementary file 5 — Significant genotypic association of variants at 11q23.3 chromosomal region with dyslipidemia under different genetic models: Odds ratios from logistic regression analyses before and after adjusting for age, sex and BMI. (DOCX 16 kb) [file 12944_2017_507_MOESM5_ESM.docx]

**Table S5 Significant genotypic association of variants at 11q23.3 chromosomal region with dyslipidemia under different genetic models: Odds ratios from logistic regression analyses before and after adjusting for age, sex and BMI**

| **SNP** | **Model** | **Genotype** | **Frequency** | | **Unadjusted** | | **Adjusted for age, sex and BMI** | |
| --- | --- | --- | --- | --- | --- | --- | --- | --- |
|  |  |  | **Cases** | **Control** | **OR (95% CI)** | **p-value** | **OR (95% CI)** | **p-value** |
| rs17440396 | Over Dominant | GG-AA | 0.54 | 0.68 | 1.7  (1.17-2.53) | 0.005 | 1.77  (1.2-2.62) | 0.0041 |
|  |  | AG | 0.46 | 0.32 |  |  |  |  |
| rs10488699 | Recessive | GG-AG | 0.96 | 0.9 | 3.2  (1.1-9.4) | 0.025 | 3.02  (1.0-9.0) | 0.039 |
|  |  | AA | 0.05 | 0.1 |  |  |  |  |
| **rs2187126*** | Dominant | AA | 0.64 | 0.82 | 2.72  (1.76-4.19) | 4.76x10^-6^ | 2.75  (1.77-4.28) | 5.15 x10^-6^ |
|  |  | AG-GG | 0.36 | 0.18 |  |  |  |  |
| rs6589566 | Log-additive | -- | -- | -- | 0.67  (0.50-0.90) | 0.006 | 0.67  (0.50-0.91) | 0.0080 |
| **rs633389*** | Dominant | CC | 0.62 | 0.79 | 2.41  (1.58-3.65) | 3.28x10^-5^ | 2.42  (1.58-3.71) | 4.14x10^-5^ |
|  |  | CC-TT | 0.38 | 0.21 |  |  |  |  |
| rs672143 | Log-additive | -- | -- | -- | 0.09  (0.01-0.69) | 0.004 | 0.09  (0.01-0.71) | 0.001 |
| **rs1263163*** | Dominant | GG | 0.44 | 0.74 | 3.55  (2.4-5.27) | 1.1x10^-10^ | 3.63  (2.43-5.27) | 1.1x10^-10^ |
|  |  | GA-AA | 0.56 | 0.26 |  |  |  |  |
| rs1263171 | Recessive | GG-AG | 0.73 | 0.82 | 1.77  (1.22-2.78) | 0.013 | 1.80  (1.14-2.86) | 0.012 |
|  |  | AA | 0.27 | 0.18 |  |  |  |  |
| rs2854116 | Recessive | GG-AG | 0.84 | 0.72 | 0.49  (0.29-0.82) | 0.005 | 0.46  (0.27-0.79) | 0.003 |
|  |  | AA | 0.16 | 0.28 |  |  |  |  |
| **rs632153*** | Dominant | AG-GG | 0.62 | 0.41 | 2.38  (1.78-3.15) | 6.04x10^-10^ | 2.5  (1.75-3.44) | 9.71x10^-8^ |
|  |  | AA | 0.38 | 0.59 |  |  |  |  |

**^*^**Significant after correction for multiple testing
