# Supplementary material for: Quantitative trait loci at the 11q23.3 chromosomal region related to dyslipidemia in the population of Andhra Pradesh, India
Source: Lipids Health Dis. 2017 Jun 13;16:116. doi: 10.1186/s12944-017-0507-5 (PMC5470178; doi:10.1186/s12944-017-0507-5)
Supplement: Supplementary file 6 — Percentage of Dyslipidemic and Non dyslipidemic individuals with cumulative risk scores for SNPs associated with dyslipidemia and results of logistic regression analysis of dyslipidemia on risk categories. (DOCX 11 kb) [file 12944_2017_507_MOESM6_ESM.docx]

**Table S6 Percentage of Dyslipidemic and Non dyslipidemic individuals with cumulative risk scores for SNPs associated with dyslipidemia and results of logistic regression analysis of dyslipidemia on risk categories**

| **Risk Category** | **Risk Score** | **Percentage of Dyslipidemic Individuals** | **Percentage of Non dyslipidemic Individuals** | **OR (95% CI)** | **Z value** | **p value** |
| --- | --- | --- | --- | --- | --- | --- |
| 1 | 2-4.9 | 5.5 | 18.6 | Reference |  |  |
| 2 | 5-5.9 | 10.9 | 30.6 | 1.3 (0.4-4.4) | 0.45 | 0.650 |
| 3 | 6-6.9 | 21.9 | 42.1 | 1.8 (0.6-5.7) | 1.1 | 0.200 |
| 4 | 7-7.9 | 21.9 | 18.6 | 4.1 (1.2-13.3) | 2.4 | 0.016* |
| 5 | 8-8.9 | 13.7 | 11.5 | 4.5 (1.2-16.2) | 2.3 | 0.018* |
| 6 | 9-9.9 | 9.8 | 5.5 | 6.0 (1.5-24.7) | 2.4 | 0.013* |
| 7 | 10-10.9 | 10.4 | 6.0 | 6.0 (1.5-24.7) | 2.4 | 0.013* |
| 8 | 11-14.29 | 6.0 | 2.2 | 10.8 (1.6-71) | 2.4 | 0.013* |

* Significant p value
