# Supplementary figures and images for: Quantitative trait loci at the 11q23.3 chromosomal region related to dyslipidemia in the population of Andhra Pradesh, India
Source: Lipids Health Dis. 2017 Jun 13;16:116. doi: 10.1186/s12944-017-0507-5 (PMC5470178; doi:10.1186/s12944-017-0507-5)

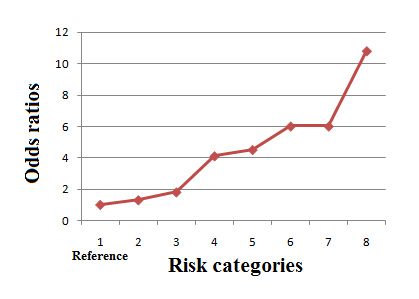

Supplement: Supplementary file 7 — Plot of Odds ratios for cumulative risk score categories with reference to baseline category 1. (JPEG 28 kb) [file 12944_2017_507_MOESM7_ESM.jpg]
