# Supplementary material for: Quantitative trait loci at the 11q23.3 chromosomal region related to dyslipidemia in the population of Andhra Pradesh, India
Source: Lipids Health Dis. 2017 Jun 13;16:116. doi: 10.1186/s12944-017-0507-5 (PMC5470178; doi:10.1186/s12944-017-0507-5)
Supplement: Supplementary file 8 — Results of pair wise SNP-SNP interaction analysis; only significant pairs are represented in the table. (DOCX 11 kb) [file 12944_2017_507_MOESM8_ESM.docx]

**Table S7 Results of pair wise SNP-SNP interaction analysis; only significant pairs are represented in the table.**

| **SNP Pair** | | **Gene** | **VLDL** | | **TG** | |
| --- | --- | --- | --- | --- | --- | --- |
| **SNP1** | **SNP2** |  | **Β** | **p-value** | **Β** | **p-value** |
| rs3741300 | rs5132 | *BUD13*-*APOC3* | 33.44 | 1.14 x10^-5^ | 168.4 | 1.00 x10^-5^ |
|  | rs5081 | *BUD13*-*APOA1* | 32.26 | 9.37 x10^-5^ |  |  |
| rs603446 | rs5081 | *ZPR1*-*APOA1* | 34.96 | 3.27 x10^-5^ | 173.6 | 3.79 x10^-5^ |
|  | rs632153 | *ZPR1*-*APOA1* | 34.15 | 7.36 x10^-5^ | 169.1 | 8.78 x10^-5^ |
| rs1787680 | rs5081 | *APOA5*-*APOA1* | 34.72 | 3.75 x10^-5^ | 172.4 | 4.31 x10^-5^ |
|  | rs632153 | *APOA5*-*APOA1* | 33.88 | 8.50 x10^-5^ |  |  |
| rs2854116 | rs5081 | *APOC3*-*APOA1* | 39.03 | 5.60 x10^-5^ | 195.2 | 5.74 x10^-5^ |

β linear regression coefficient, Blank cell – not significant
